# The Essential Role of SIRT1 in Hypothalamic-Pituitary Axis

**DOI:** 10.3389/fendo.2018.00605

**Published:** 2018-10-23

**Authors:** Masaaki Yamamoto, Yutaka Takahashi

**Affiliations:** ^1^Pituitary Center, Department of Medicine, Cedars-Sinai Medical Center, Los Angeles, CA, United States; ^2^Division of Diabetes and Endocrinology, Department of Internal Medicine, Kobe University Graduate School of Medicine, Kobe, Japan

**Keywords:** SIRT1, adaptation, calorie restriction, hypothalamus, pituitary

## Abstract

The endocrine system plays an essential role in the physiological adaptation to malnutrition. The adaptive response of various hormones directs the energy utilization toward the survival functions and away from growth and reproduction. Particularly, the hypothalamic pituitary axis plays an integral and a central role in the regulation of endocrine organs. Sirtuin 1 (SIRT1) is a nicotinamide adenine dinucleotide (NAD)-dependent histone deacetylase that is activated in response to calorie restriction (CR). SIRT1 is involved in cellular processes via the deacetylation of histone as well as various transcription factors and signal transduction molecules and thereby modulates the endocrine/metabolic functions. There is much evidence to demonstrate clearly that SIRT1 in the hypothalamus, pituitary gland, and other target organs modifies the synthesis, secretion, and activities of hormones and in turn induces the adaptive responses. In this review, we discussed the role of SIRT1 in the hypothalamic pituitary axis and its pathophysiological significance.

## Introduction

### The physiological role of sirtuin 1 (SIRT1)

SIR2 is the first sirtuin protein to be discovered in *Saccharomyces cerevisae* (yeast) (1). SIR2 orthologs are highly conserved throughout various species including mammals, plants, bacteria, worms, flies, and fishes. In mammals, the seven SIR2 orthologs are termed SIRT1-7 and are presumed to be ubiquitously expressed in all the tissues (2). Sirtuins were initially reported as protein deacetylases and ADP ribosyltransferases (3, 4). However, subsequent analyses revealed that sirtuins exhibit various enzymatic activities. For instance, SIRT5 exhibits lysine-desuccinylase and -demalonylase activities (5, 6). These enzymatic activities of sirtuins are generally activated in response to CR by the utilization of nicotine adenine dinucleotide (NAD+) as the co-substrate.

Regarding the intracellular localization of sirtuins, SIRT1, SIRT6, and SIRT7 are localized in the nucleus and mostly regulates protein function by the post-translational modification via histone deacetylation (7). Moreover, SIRT1 localizes in the cytosol and directly deacetylates various transcription factors and cofactors. SIRT2 is localized in the cytosol and nucleus and is involved in the metabolic process and cell cycle regulation (8–11). SIRT3, SIRT4, and SIRT5 are localized in the mitochondria and regulate various metabolic enzyme activities and mitochondrial oxidative stress (9). Therefore, in the organisms, sirtuins coordinate the cellular responses to adapt to CR in their corresponding cellular compartments in which they occur.

Among the seven sirtuins, SIRT1 is the most studied and well-characterized sirtuin with respect to its physiological functions. SIRT1 is involved in the production of various hormones and maintenance of homeostasis by regulating the function of histones and transcription factors, to adapt to malnutrition in the endocrine and metabolic systems. For instance, SIRT1 modulates forkhead box protein O1 (FOXO1), peroxisome proliferator-activated receptor gamma coactivator 1-alpha (PGC-1α), signal transducer and activator of transcription (STAT3) (12–14). SIRT1 interacts with peroxisome proliferator-activated receptor (PPARγ) and represses its transcriptional activity to reduce adipogenesis in differentiated fat cells and mobilize free fatty acid from the white adipose tissues (15). Additionally, SIRT1 negatively regulates the mitochondrial uncoupling protein 2 (UCP2) expression and enhances the insulin secretion in response to glucose level elevation in the pancreas (16).

Recently, accumulating evidence suggests that SIRT1 plays important role in the homeostasis maintenance in the neuroendocrine system. Particularly, the hypothalamic pituitary axes play a central and an integral role in the neuroendocrine system.

## Physiology of the hypothalamus and pituitary gland

The neuroendocrine system, especially the hypothalamus and pituitary gland play an essential role in the homeostasis maintenance via orchestrating the endocrine system as a regulatory machinery. The hypothalamus–pituitary complex connects the nervous system with the endocrine system that regulates the systemic hormone secretion in a direct or indirect manner. The neural signaling of central nervous system modifies the hypothalamus–pituitary axis and results in the regulation of corresponding target hormonal secretion (17).

The hypothalamus is responsible for the homeostasis maintenance by regulating the body temperature, appetite, thirst, energy expenditure, behavior, and circadian rhythm of the organisms (18). Moreover, the hypothalamus synthesizes and secretes various hypothalamic hormones that in turn stimulate or inhibit the secretion of pituitary hormones (19). The medial preoptic nucleus regulates the release of gonadotropic hormones and thermogenesis (20). The putative anterior paraventricular (aPV), the paraventricular nucleus (PVN), and the supraoptic nucleus (SON) consist of parvicellular neurons that release corticotropin-releasing hormone (CRH), thyrotropin-releasing hormone (TRH), growth hormone releasing hormone (GHRH), and somatostatin (SST), and magnocellular neurons that produce vasopressin (AVP) and oxytocin (OXT) (21–24).

The pituitary gland consists of anterior posterior lobes and the anterior lobe is functionally subdivided into six cell types based on the production of a specific hormone by each cell type (25). The growth hormone (GH), prolactin, adrenocorticotropic hormone (ACTH), thyroid stimulating hormone (TSH), and luteinizing hormone (LH) or follicular stimulating hormone (FSH) are secreted by the somatotroph, lactotroph, corticotroph, thyrotroph, and gonadotroph, respectively (26). The anterior pituitary hormones are mainly regulated by the release hormones or inhibitory hormones from the hypothalamus via the portal hypophyseal circulation into the anterior lobe and neural connection to the posterior lobe through the pituitary stalk (a physical and functional connection between the hypothalamus and pituitary gland) (27, 28). The hormones secreted by the pituitary gland circulate in the systemic blood flow and thereby stimulate the hormone secretion by each of their respective target organ. Generally, these pathways initiating from the hypothalamus to target organ via the pituitary gland are mainly classified into four axes depending on the target organ, namely, hypothalamus-pituitary-adrenal (HPA), hypothalamus-pituitary-thyroid (HPT), hypothalamus-pituitary-gonadal (HPG), and somatotropic axes. In this review, we described the SIRT1 involvement in each of the aforementioned axes in the subsequent subsections.

### SIRT1 in hypothalamus

In the hypothalamus, SIRT1 is expressed in the SF1 neuron in the ventromedial hypothalamic nucleus (VMH) and in the POMC and AgRP neuron in the arcuate nucleus (ARH), respectively (29–31). POMC neuron specific SIRT1 knockout mice exhibited energy imbalance due to the altered sympathetic activity (30). Also, deletion of SIRT1 in SF1 neurons caused insulin resistance in skeletal muscle. On the contrary, SIRT1 overexpression in SF1 neurons prevented from diet-induced obesity and insulin resistance (29). Both targeted overexpression of SIRT1 in POMC or AgRP neurons prevented age-associated weight gain. However, SIRT1 overexpression in POMC neurons enhanced the energy expenditure mediated by increment of sympathetic activity in adipose tissue while SIRT1 overexpression in AgRP neurons suppressed food intake, indicating a presence of nuclear specific mechanisms (31). Also, food restriction increases SIRT1 protein levels in the dorsomedial (DMH) and lateral hypothalamic nuclei (LH) (32). Brain specific SIRT1 overexpressing mice exhibited extended life span mediated by enhanced neural activity in DMH and LH, through increased orexin type 2 receptor (Ox2r) expression (32). These data indicate that region specific expression of SIRT1 plays an important role in the regulation of appetite, energy expenditure, general metabolism, and life span.

### The HPA axis

The HPA axis is an important neuroendocrine system that plays an essential role in the survival, stress response, metabolism, appetite, immunoreaction, mood, and behavior of the organisms. Corticotrophin releasing hormone (CRH) secreted by the PVN in the hypothalamus initiates stress response in the HPA axis via the production of proopiomelanocortin (POMC) by the pituitary corticotroph. POMC is a precursor polypeptide of ACTH and alpha-melanocyte stimulating hormone (αMSH) that are cleaved by prohormone convertases 1 and 2 (PC1 and PC2), respectively. ACTH induced by corticotroph stimulates the glucocorticoid production by the adrenal cortex.

The SIRT1 expression in the hypothalamic POMC neuron is enhanced by fasting and is associated with the reduced αMSH levels, although the significance to the HPA axis is unknown (33). On the other hand, another group reported that POMC neuron specific ablation of SIRT1 did not alter POMC, ACTH, and αMSH levels (30). The precursor pro-CRH is post-translationally processed by the PC1 and PC2. SIRT1 increases the PC2 levels in the PVN that in turn increases the active-CRH production and results in the HPA axis activation (34). In mouse corticotroph cell line (AtT20 cells), treatment with resveratrol (a SIRT1 activating compound) increased the PC1 and PC2 levels. In accordance with this, Ex-527 (a SIRT1 specific inhibitor) treatment decreased the PC1 and PC2 levels in AtT20 cells (33). Although there have been no reports demonstrating the expression of SIRT1 in the corticotrophs, these data suggest that SIRT1 may indirectly modulates the HPA axis by regulating the PC1 and PC2 levels. Additionally, resveratrol increased the expression and prolonged the half-life of P450 side chain cleavage enzyme (P450scc) in the adrenal gland that results in an increase in glucocorticoid secretion by the adrenal cortex (35). Moreover, the hypothalamic SIRT1 plays an important role in the energy homeostasis maintenance associated with the HPA axis. These data clearly indicate that SIRT1 in the hypothalamus, pituitary, and adrenal gland regulates the HPA axis activation that acts as an adaptive response to starvation (Table 1A).

**Table 1:** The role of SIRT1 in hypothalamic-pituitary axis.

	**A**		**B**		**C**		**D**	
	**HPA axis**		**HPT axis**		**HPG axis**		**Somatotrophic axis**	
SIRT1	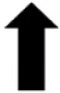	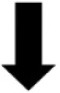	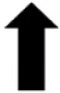	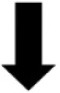	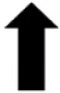	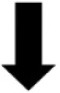	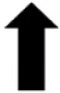	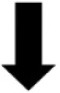
Hypothalamus	PC2 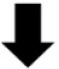 CRH 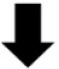	POMC 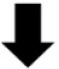				GnRH 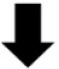		
Pituitary	a-MSH 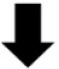 PC1/ 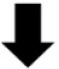 PC2	PC1/PC2 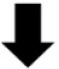	TSH 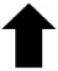			LH 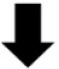 FSH 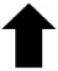	GH 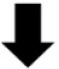	GH 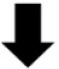
Periphery	AdrenalGC 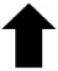			ThyroidT4 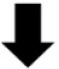		Testis Testosterone 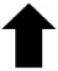	IGF-I 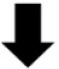	IGF-I 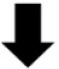

*(A) HPA axis, (B) HPT axis, (C) HPG axis, (D) somatotrophic axis*.

*SIRT1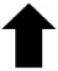, Overexpression/activation of SIRT1. SIRT1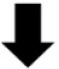, Knockout/inhibition of SIRT1. GC, Glucocorticoid, T4, Thyroxine*.

### The HPT axis

The HPT axis is activated by the thyrotropin-releasing hormone (TRH) secretion by the hypothalamus when the hypothalamus senses low levels of circulating thyroid hormone. The TRH induces thyroid-stimulating hormone (TSH) secretion by thyrotroph cells in the pituitary that in turn stimulates the thyroid hormone (TH) secretion by the thyroid to maintain normal TH levels (36). SIRT1 is expressed in thyrotroph cells and enhances TSH endocytosis via the deacetylation of phosphatidylinositol-4-phosphate 5-kinase type 1γ (PIP5K1γ), which is a main enzyme that synthesizes phosphatidylinositol 4,5-bisphosphate in thyrotroph cells (37). Tyrotroph-specific SIRT1 knockout mice showed decreased TSH secretion resulting in decreased metabolic rate (37). Additionally, SIRT1 knockout in whole body exhibited hypermetabolism caused by an increased oxygen consumption in the hepatic mitochondria leading to the decreased body weight while the mice manifested hyperphagia, reduced serum thyroxin level, and decreased nocturnal physical activity (38). Interestingly, thyroxin treatment suppressed the fasting-induced SIRT1 expression as thyroxin negatively regulates the SIRT1 level and activity in the liver via TH receptor-β (39). These data indicate that the HPT axis and SIRT1 interact with each other and adaptively regulate metabolism (Table 1B).

### The HPG axis

The HPG axis is responsible for the reproduction, life cycle, and sexual dimorphism in the organisms. Gonadotropin-releasing hormone (GnRH) is secreted by GnRH neurons that diffusely localize and form a network named pulse-generator in the hypothalamus. The pulsatile secretion of GnRH stimulates the luteinizing hormone (LH) and follicle-stimulating hormone (FSH) secretion by pituitary gonadotroph cells and subsequently the gonads produce estrogen or testosterone (36, 40).

Although secondary effect of the general condition including body weight loss cannot be ruled out, SIRT1 knockout mice exhibited a diminished hypothalamic GnRH expression and in turn reduced serum LH and FSH levels and spermatogenesis arrest, suggesting an important role of SIRT1 in the HPG axis (41). Additionally, GnRH treatment decreases SIRT1 level via the miR-132/212 induction in the pituitary. This results in the downregulation of SIRT1-dependent FOXO1 deacetylation and a decrease in the FOXO1-mediated inhibition of Fshβ transcription that ultimately increases the Fshβ expression in rat primary pituitary cells and LβT2 cell line (42) (Table 1C).

### The somatotropic axis

In the somatotropic axis, GH releasing hormone (GHRH) that is secreted by the hypothalamus induces the GH secretion by pituitary somatotroph cells. Circulating GH binds to GH receptor on hepatocytes and results in the increased serum insulin like growth factor-I (IGF-I) level (36). Moreover, GH induces local IGF-I production in various tissues including bone, muscle, and fat tissue. In various species, numerous evidences demonstrated that the reduced function of somatotropic axis extends lifespan in animal models (43–48). Regarding the underlying mechanisms, among the species which evolved from C. elegans to mouse, an evolutionarily conserved interplay between SIR2/SIRT1 and the somatotropic axis was reported in which SIR2/SIRT1 modulates the signaling molecules of somatotropic pathway (49–54).

SIRT1 brain-specific knockout (BSKO) mice exhibited dwarfism with small pituitary and reduced GH-IGF-I levels while the other pituitary hormonal levels were unaltered (55). Interestingly, SIRT6 BSKO manifested similar phenotype as that of SIRT1 BSKO. Despite the significant reduction in the number of somatotroph cells and GH content in the pituitary gland of SIRT6 BSKO, the hypothalamic GHRH and somatotropin release–inhibiting factor (SRIF) levels remained unaltered (56). Although the precise mechanism remains unclear in these models, it is speculated that aberrations during the pituitary somatotroph development and GH synthesis were presumably caused by the hypothalamic dysfunction or feedback dysregulation between the hypothalamus and pituitary gland.

Moreover, the direct role of SIRT1 in pituitary somatotroph cells was reported (57). The SIRT1 activation in somatotroph cells suppressed the GHRH-induced GH secretion in *in vivo* and *in vitro* studies. SIRT1 deacetylates glycogen synthase kinase 3 beta (GSK3β) and cAMP response element-binding protein (CREB). Deacetylated-GSK3β gets activated and inactivates CREB via protein phosphatase-1. Deacetylated-CREB exhibits a decrease in its activity and is unable to activate the transcription of POU domain, class 1, transcription factor 1 that results in the GH synthesis impairment (57). These data demonstrate that SIRT1 regulates the somatotropic axis in the hypothalamus and pituitary gland.

**Figure 1:**
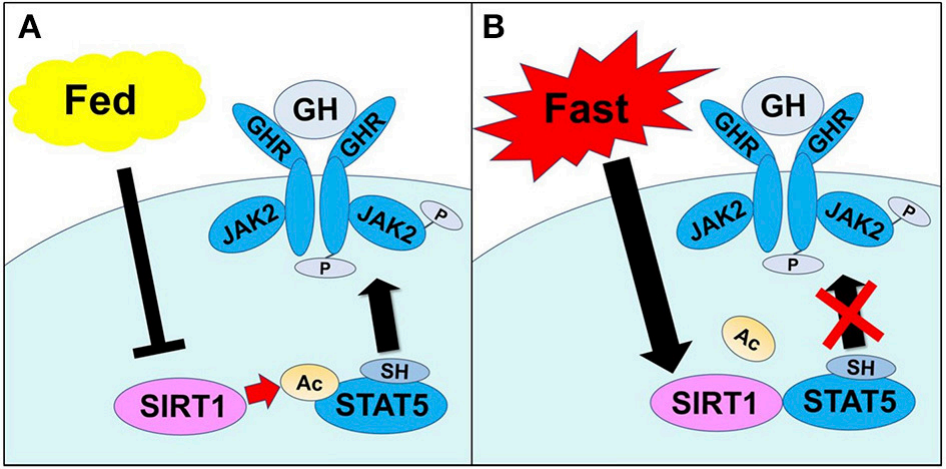
The mechanisms through which SIRT1 regulates STAT5 activation by GH. **(A)** In the fed condition, the SH2 domain of STAT5 recognizes and binds to Tyr-phosphorylated GHR, causing JAK2 to phosphorylate and activate STAT5. **(B)** In the fasting condition, SIRT1 is activated and interacts with STAT5, thereby deacetylating Lys residues adjacent to the SH2 domain of STAT5. This results in an impaired ability to bind Tyr-phosphorylated GHR, which inhibits activation of STAT5. Excerpt from, Yamamoto et al. (60).

During starvation condition, it is well-known that the reduced serum IGF-I level is observed despite the elevated GH level. Moreover, it is reported that exogenous GH treatment did not increase the serum IGF-I level in starving individuals (58). This is considered as GH resistance status in the liver (59). In the organisms, it is considered that GH resistance is an adaptive response to survive during starvation and malnutrition conditions. These responses include the decreased IGF-I level inhibits growth and the elevated GH level causes insulin resistance and free fatty acid mobilization to avoid hypoglycemia. Therefore, we hypothesized that SIRT1 might negatively regulate the GH-dependent IGF-I production during starved condition in the liver. We demonstrated that hepatic SIRT1 directly deacetylates STAT5 and suppresses the tyrosine phosphorylation of STAT5 via GH that results in the impairment of GH signaling during fasting condition (60) (Figure 1).

Considering that the somatotropic axis utilizes energy to promote body growth, SIRT1 signaling basically utilizes energy to improve the survival of organisms during CR. Conclusively, SIRT1 plays an important role in switching from growth to survival mode in order to adapt to malnutrition by modulating the somatotropic axis at various steps (Table 1D).

## Conclusion

These studies clearly demonstrated that SIRT1 is involved in the regulatory mechanism of hypothalamus-pituitary axis with respect to the homeostasis maintenance. The determination of a crosstalk between the neuroendocrine system and SIRT1 function is crucial as both of them play an important role in the homeostasis maintenance as well as the regulation of lifespan.

## Author contributions

MY drafted the manuscript and MY and YT were responsible for the conception and design of this review.

### Conflict of interest statement

The authors declare that the research was conducted in the absence of any commercial or financial relationships that could be construed as a potential conflict of interest.
